# Interactive effects of platelet-to-lymphocyte ratio and FIB-4 index for risk stratification of liver fibrosis in metabolic dysfunction-associated steatotic liver disease: performance assessment comparing interactive models with traditional composite scores

**DOI:** 10.3389/fmed.2026.1804716

**Published:** 2026-05-13

**Authors:** Mei Shi, Guoliang Zhang, Hao Zhou, Yan Li, Yong Hou, Xiaojun Yang, Lili Liu, Guangdong Tong

**Affiliations:** 1 Shenzhen Traditional Chinese Medicine Hospital Affiliated to Nanjing University of Chinese Medicine, Shenzhen, China; 2 Department of Infectious Diseases, The First Affiliated Hospital of Anhui University of Chinese Medicine, Hefei, Anhui, China; 3 Department of Liver Disease, Shenzhen Traditional Chinese Medicine Hospital, Shenzhen, China

**Keywords:** FIB-4 index, hepatic fibrosis, interaction, non-alcoholic fatty liver disease, platelet-to-lymphocyte ratio, predictive model

## Abstract

**Background:**

Metabolic Dysfunction-Associated Steatotic Liver Disease (MASLD) is the most common chronic liver disease globally, and the accurate assessment of hepatic fibrosis is crucial for guiding therapeutic decisions. This study aimed to investigate the diagnostic value of the interaction between the platelet-to-lymphocyte ratio (PLR) and the Fibrosis-4 (FIB-4) index for predicting significant hepatic fibrosis in patients with MASLD.

**Methods:**

This retrospective study analyzed 358 MASLD patients who underwent liver biopsy at two university-affiliated hospitals between January 2020 and December 2024. Based on histopathology, significant fibrosis was defined as fibrosis stage ≥F2. Four predictive models were constructed: PLR alone, FIB-4 alone, a combined PLR + FIB-4 model, and a PLR × FIB-4 interaction model. Model performance was evaluated using the area under the receiver operating characteristic curve (AUC), net reclassification index (NRI), and integrated discrimination improvement (IDI), and compared against traditional non-invasive scores.

**Results:**

Among the 358 patients, 168 (46.9%) had significant fibrosis. PLR was positively correlated with the FIB-4 index (*r* = 0.542, *p* < 0.001), and this correlation strengthened with increasing fibrosis severity. The interaction model demonstrated the best diagnostic performance, yielding an AUC of 0.824 (95% CI: 0.778–0.870) for significant fibrosis, with 78.6% sensitivity and 78.9% specificity. For predicting advanced fibrosis (≥F3), its AUC was 0.852 (95% CI: 0.803–0.901). Compared to the APRI-FIB-4 composite score, the interaction model significantly improved reclassification (NRI: 0.142, *p* = 0.008) and discrimination (IDI: 0.067, *p* = 0.003). A three-tier risk stratification strategy based on the model also showed excellent clinical utility (*p* for trend <0.001). Bootstrap internal validation (*n* = 1,000) confirmed the model’s stability and calibration.

**Conclusion:**

The PLR × FIB-4 interaction model is a novel and effective tool for the non-invasive assessment of hepatic fibrosis in patients with MASLD. This model significantly outperforms individual markers and traditional scoring systems and demonstrates excellent risk stratification capability, holding significant promise for supporting individualized clinical management.

## 1 Introduction

Non-alcoholic fatty liver disease (MASLD) is the most prevalent chronic liver disease globally, affecting 25–30% of the adult population. Its incidence is rising in parallel with the epidemics of obesity and diabetes (1). MASLD encompasses a wide spectrum of conditions, ranging from simple steatosis to non-alcoholic steatohepatitis (NASH), hepatic fibrosis, cirrhosis, and ultimately, hepatocellular carcinoma (2). Among these, the accurate assessment of hepatic fibrosis is critical for staging, prognosis, and therapeutic decision-making. Significant fibrosis (≥F2) substantially increases the risk of liver-related mortality, while advanced fibrosis (≥F3) is an independent risk factor for liver transplantation and hepatocellular carcinoma (3).

Although liver biopsy remains the gold standard for evaluating hepatic fibrosis, its invasive nature, potential for sampling error, and risk of complications limit its widespread clinical use (4). Consequently, developing accurate, convenient, and cost-effective non-invasive assessment methods has become a research priority (5). Currently available non-invasive tools include the aspartate aminotransferase-to-platelet ratio index (APRI), the Fibrosis-4 (FIB-4) index, the MASLD Fibrosis Score (NFS), and the BARD score (6). However, the diagnostic performance of these traditional scores is often suboptimal in MASLD populations, leaving a significant “gray zone,” particularly in distinguishing intermediate stages of fibrosis (7).

The role of inflammation in MASLD progression has garnered increasing attention (8). The platelet-to-lymphocyte ratio (PLR), a readily available marker of systemic inflammation and platelet dynamics, has shown promise for predicting fibrosis in various chronic liver diseases (9). In this context, thrombocytopenia can indicate portal hypertension and hypersplenism, while lymphopenia often reflects immune dysfunction in chronic inflammatory states (10). By integrating these parameters, the PLR may capture the complex pathophysiology of hepatic fibrosis more accurately than either marker alone.

The FIB-4 index, which incorporates age, platelet count, and liver enzymes, is one of the most widely used non-invasive tools for fibrosis assessment (11). Numerous large-scale studies have validated its predictive value in patients with MASLD, demonstrating relatively stable performance across diverse populations (12). However, individual scoring systems often fail to capture the complex mechanisms underlying hepatic fibrosis, especially the potential interactions among different biomarkers (13).

Biomarker interactions refer to the non-additive, or synergistic, effects that occur when two or more variables act in combination, potentially enhancing the performance of predictive models. The pathological progression of hepatic fibrosis involves complex interactions among inflammation, platelet dynamics, and hepatocellular injury (14). It is plausible that the inflammatory status reflected by PLR could modulate the relationship between the FIB-4 index and fibrosis severity, while the platelet data within FIB-4 could, in turn, influence the predictive value of PLR (15).

Traditional composite scores typically use linear-weighted methods, assuming independence among variables and thereby overlooking these potential interactions (16). In contrast, interaction models can capture non-linear relationships by introducing product terms, which may offer a more accurate reflection of complex biological processes (17). Indeed, recent studies in cardiovascular disease and oncology have shown that models incorporating biomarker interactions significantly outperform traditional additive models (18).

Currently, research investigating the interaction between the PLR and FIB-4 index for MASLD fibrosis assessment is limited. Existing studies have focused primarily on the individual predictive value of these markers, without exploring their potential synergy (19). Furthermore, these studies often have limitations related to sample size, design, and methodology, and a standardized framework for assessing biomarker interactions has not yet been established (20).

Therefore, this study aims to systematically evaluate the diagnostic value of the interaction between the PLR and FIB-4 index for hepatic fibrosis risk stratification in patients with MASLD. By constructing a predictive model that includes an interaction term and comparing it comprehensively with traditional single markers and composite scores, we aim to provide clinicians with a more precise non-invasive tool for fibrosis assessment and to establish a scientific foundation for individualized therapeutic decisions.

## 2 Materials and methods

### 2.1 Study design and participants

This retrospective study included consecutive MASLD patients who underwent liver biopsy at the First Affiliated Hospital of Anhui University of Traditional Chinese Medicine and Shenzhen Hospital of Traditional Chinese Medicine affiliated to Nanjing University of Traditional Chinese Medicine between January 2020 and December 2025. The study was approved by the hospital ethics committee and conducted in accordance with the Declaration of Helsinki. Inclusion criteria: (1) Age 18–75 years; (2) MASLD diagnosis confirmed according to AASLD guidelines (21); (3) Liver biopsy performed within 6 months of blood sampling; (4) Complete clinical and laboratory data available. Exclusion criteria: (1) Daily alcohol consumption >20 g for women or >30 g for men; (2) Coexisting viral hepatitis, autoimmune liver disease, or drug-induced liver injury; (3) Decompensated liver disease or hepatocellular carcinoma; (4) Active infection or inflammatory disease within 2 weeks; (5) Hematological disorders affecting platelet or lymphocyte counts; (6) Use of immunosuppressive agents. The specific inclusion and exclusion criteria were chosen to ensure cohort homogeneity and data integrity. The age range of 18–75 years was selected to focus on an adult population while minimizing the confounding effects of advanced age, where multiple comorbidities can influence results and scores like FIB-4 are known to have reduced accuracy. The six-month interval between blood sampling and liver biopsy was established as a clinically accepted timeframe that balances the practical challenges of retrospective research with the need to ensure that laboratory markers accurately reflect the histological stage of fibrosis, as significant progression within this period is considered minimal.

### 2.2 Data collection and parameter calculation

Baseline characteristics were systematically collected for all enrolled patients, including demographic features such as age, gender, height, weight, and BMI, as well as comorbidity information including hypertension, diabetes mellitus, and dyslipidemia. Laboratory assessments encompassed complete blood count parameters (hemoglobin, white blood cells, platelets, lymphocytes, etc.), biochemical indicators (ALT, AST, total bilirubin, albumin, and other liver function parameters), and metabolism-related markers. Laboratory data were obtained from a hospital certified clinical laboratory. Ensure comparability and reliability of measurements. A complete case analysis approach was used in this study; any patients with missing data on variables required for model building were excluded from the final analysis. Outliers will not be systematically removed unless confirmed as data entry errors during data validation. All blood samples were collected in the early morning following a 12-h fasting period to ensure standardization and comparability of test results. Based on laboratory test results, non-invasive hepatic fibrosis assessment indices required for the study were calculated using standard formulas: PLR (platelet-to-lymphocyte ratio) = platelet count (×10^9^/L) / lymphocyte count (×10^9^/L); FIB-4 index = [age (years) × AST (U/L)] / [platelet count (×10^9^/L) × √ALT (U/L)]; APRI index = [AST (U/L) / upper limit of normal for AST] / [platelet count (×10^9^/L)] × 100. All calculations employed unified standardized procedures to ensure data quality and consistency.

### 2.3 Histological assessment

Percutaneous liver biopsies were performed on all patients using a standardized 16-gauge needle to obtain adequate hepatic tissue specimens (≥15 mm in length). The specimens were fixed in 10% neutral formalin, embedded in paraffin, and sectioned at 4 μm. Sections were then stained with hematoxylin–eosin and Masson’s trichrome to evaluate hepatic inflammatory activity and fibrosis severity. All histological assessments were uniformly performed by experienced hepatopathologists specializing in MASLD diagnosis. Inflammatory activity was graded using the MASLD Activity Score (NAS), and fibrosis was staged according to the Kleiner criteria (22): F0, no fibrosis; F1, perisinusoidal fibrosis; F2, portal and perisinusoidal fibrosis; F3, bridging fibrosis; and F4, cirrhosis. For this study, significant fibrosis was defined as stage ≥F2 and advanced fibrosis as stage ≥F3. These definitions served as the pathological gold standard for our analysis.

### 2.4 Statistical analysis

Statistical analyses were conducted using SPSS version 26.0 and R version 4.3.0. Continuous variables were presented as mean ± standard deviation (SD) for normally distributed data or median [interquartile range (IQR)] for non-normally distributed data. Categorical variables were expressed as frequencies and percentages. Group comparisons were made using the Student’s t-test, Mann–Whitney U test, or chi-square test, as appropriate for the data type and distribution. To evaluate the predictive performance of different biomarker combinations, we constructed four multivariate logistic regression models: (1) PLR alone model, assessing the independent predictive value of platelet-to-lymphocyte ratio; (2) FIB-4 alone model, evaluating the independent predictive capacity of the fibrosis index; (3) PLR + FIB-4 additive model, exploring the predictive effect of combined application of both indicators; (4) PLR × FIB-4 interaction model, examining the interaction effects between the two indicators through inclusion of the PLR × FIB-4 product term.

To address potential multicollinearity arising from the shared platelet count component in both PLR and FIB-4, specific diagnostic and preventative measures were taken. Prior to constructing the interaction model, the continuous predictors (PLR and FIB-4) were mean-centered by subtracting their respective means. This standard procedure minimizes the correlation between the main effects and their product term, thereby enhancing the stability and interpretability of the model coefficients. Subsequently, collinearity diagnostics were performed for the final model by calculating the variance inflation factor (VIF) for each predictor. The VIF values for the centered main effects and the interaction term were all found to be below 3.0, confirming that multicollinearity was not a significant concern and did not compromise the reliability of the model’s estimated coefficients. The area under the receiver operating characteristic curve (AUROC) served as the primary evaluation metric, with sensitivity, specificity, positive predictive value (PPV), negative predictive value (NPV), accuracy, and Youden index calculated to comprehensively assess the diagnostic performance of the models.

DeLong test was employed to compare statistical differences in AUROC between different models, while net reclassification index (NRI) and integrated discrimination improvement (IDI) were used to quantitatively evaluate the improvement of composite models relative to single-indicator models. To avoid overfitting and assess model stability, Bootstrap resampling (*n* = 1,000) was performed for internal validation, calculating bias-corrected confidence intervals. Hosmer-Lemeshow goodness-of-fit test was utilized to evaluate model calibration, with calibration curves plotted to visually demonstrate concordance between predicted probabilities and actual incidence rates. Based on tertiles of predicted probabilities from the optimal model, study subjects were stratified into low-, intermediate-, and high-risk groups to analyze differences in outcome event rates across risk strata, providing evidence for clinical risk stratification management. Thresholds were selected to optimize clinical utility rather than based on simple statistical distribution. A low-risk group (predicted probability <0.25) was defined to achieve a high negative predictive value (NPV) for reliably ruling out significant fibrosis. A high-risk group (predicted probability >0.75) was defined to achieve a high positive predictive value (PPV) for identifying patients requiring priority specialist referral. Patients with probabilities falling between these two thresholds (0.25–0.75) were classified as the intermediate-risk group. To further assess the robustness of the optimal interaction model, a series of sensitivity analyses were conducted. First, the model’s performance was evaluated in the clinically important subgroup of patients with normal alanine aminotransferase (ALT) levels (defined as ≤40 U/L). Second, to evaluate performance across the full spectrum of fibrosis, AUCs were calculated separately for predicting significant fibrosis (≥F2), advanced fibrosis (≥F3), and cirrhosis (F4). Third, to assess the impact of the time interval between laboratory testing and biopsy, we compared the model’s AUC in patients with a short interval (≤30 days) versus a longer interval (31–180 days). Finally, the impact of platelet-influencing comorbidities was assessed by referring to the subgroup analysis based on diabetes status. All statistical tests were two-sided, with statistical significance set at *p* < 0.05.

## 3 Results

### 3.1 Baseline patient characteristics

The study cohort comprised 358 patients with histologically confirmed MASLD. The mean age was 51.2 ± 11.8 years, and 208 (58.1%) were male. Histological staging revealed that 190 patients (53.1%) had no or mild fibrosis (F0–F1), 108 (30.2%) had moderate fibrosis (F2), 45 (12.6%) had severe fibrosis (F3), and 15 (4.2%) had cirrhosis (F4). For the primary endpoint, 168 patients (46.9%) had significant fibrosis (≥F2), and 60 (16.8%) had advanced fibrosis (≥F3). Baseline characteristics stratified by the presence of significant fibrosis are presented in Table 1. Patients with significant fibrosis (≥F2) were older (53.8 ± 12.0 vs. 48.9 ± 11.2 years; *p* < 0.001) and had a higher body mass index (29.1 ± 4.5 vs. 27.8 ± 4.0 kg/m^2^; *p* = 0.003) compared to those with no or mild fibrosis (F0–F1). The prevalence of type 2 diabetes was also significantly higher in the significant fibrosis group (47.0% vs. 33.2%; *p* = 0.007).

**Table 1:** Baseline characteristics of study participants.

Variables	Total (*n* = 358)	F0–F1 (*n* = 190)	*F* ≥ 2 (*n* = 168)	*p*-value
Demographic characteristics				
Age (years)	51.2 ± 11.8	48.9 ± 11.2	53.8 ± 12.0	<0.001
Male, *n* (%)	208 (58.1)	105 (55.3)	103 (61.3)	0.235
BMI (kg/m^2^)	28.4 ± 4.3	27.8 ± 4.0	29.1 ± 4.5	0.003
Comorbidities				
Diabetes, *n* (%)	142 (39.7)	63 (33.2)	79 (47.0)	0.007
Hypertension, *n* (%)	189 (52.8)	92 (48.4)	97 (57.7)	0.072
Dyslipidemia, *n* (%)	226 (63.1)	115 (60.5)	111 (66.1)	0.271
Laboratory parameters				
ALT (U/L)	72.5 ± 48.2	61.4 ± 40.1	85.2 ± 53.8	<0.001
AST (U/L)	58.9 ± 35.4	47.2 ± 26.3	72.3 ± 40.2	<0.001
GGT (U/L)	95.3 ± 72.1	78.6 ± 58.9	114.5 ± 82.1	<0.001
Albumin (g/L)	41.8 ± 4.9	42.9 ± 4.2	40.5 ± 5.4	<0.001
Total bilirubin (μmol/L)	16.9 ± 8.7	15.2 ± 7.4	18.9 ± 9.6	<0.001
Platelet count (×10^9^/L)	234.6 ± 62.8	251.2 ± 57.3	215.8 ± 64.9	<0.001
Lymphocyte count (×10^9^/L)	1.68 ± 0.54	1.85 ± 0.52	1.48 ± 0.52	<0.001
Calculated indices				
PLR	155.8 ± 58.4	143.2 ± 48.6	170.5 ± 65.2	<0.001
FIB-4 index	1.89 ± 1.24	1.35 ± 0.71	2.51 ± 1.48	<0.001
APRI score	1.15 ± 0.98	0.84 ± 0.64	1.51 ± 1.18	<0.001

Data are presented as mean ± SD or *n* (%). BMI, body mass index; ALT, alanine aminotransferase; AST, aspartate aminotransferase; GGT, gamma-glutamyl transpeptidase; PLR, platelet/lymphocyte ratio; APRI, AST to platelet ratio index.

Compared to the mild fibrosis group, patients with significant fibrosis had significantly higher liver enzymes, including ALT (85.2 ± 53.8 vs. 61.4 ± 40.1 U/L), AST (72.3 ± 40.2 vs. 47.2 ± 26.3 U/L), and GGT (114.5 ± 82.1 vs. 78.6 ± 58.9 U/L; all *p* < 0.001). Hematological parameters also differed significantly; the significant fibrosis group had a lower platelet count (215.8 ± 64.9 vs. 251.2 ± 57.3 × 10^9^/L) and a lower lymphocyte count (1.48 ± 0.52 vs. 1.85 ± 0.52 × 10^9^/L). Consequently, both the PLR (170.5 ± 65.2 vs. 143.2 ± 48.6) and the FIB-4 index (2.51 ± 1.48 vs. 1.35 ± 0.71) were significantly higher in this group (all *p* < 0.001).

### 3.2 Index correlation and interaction analysis

**Figure 1:**
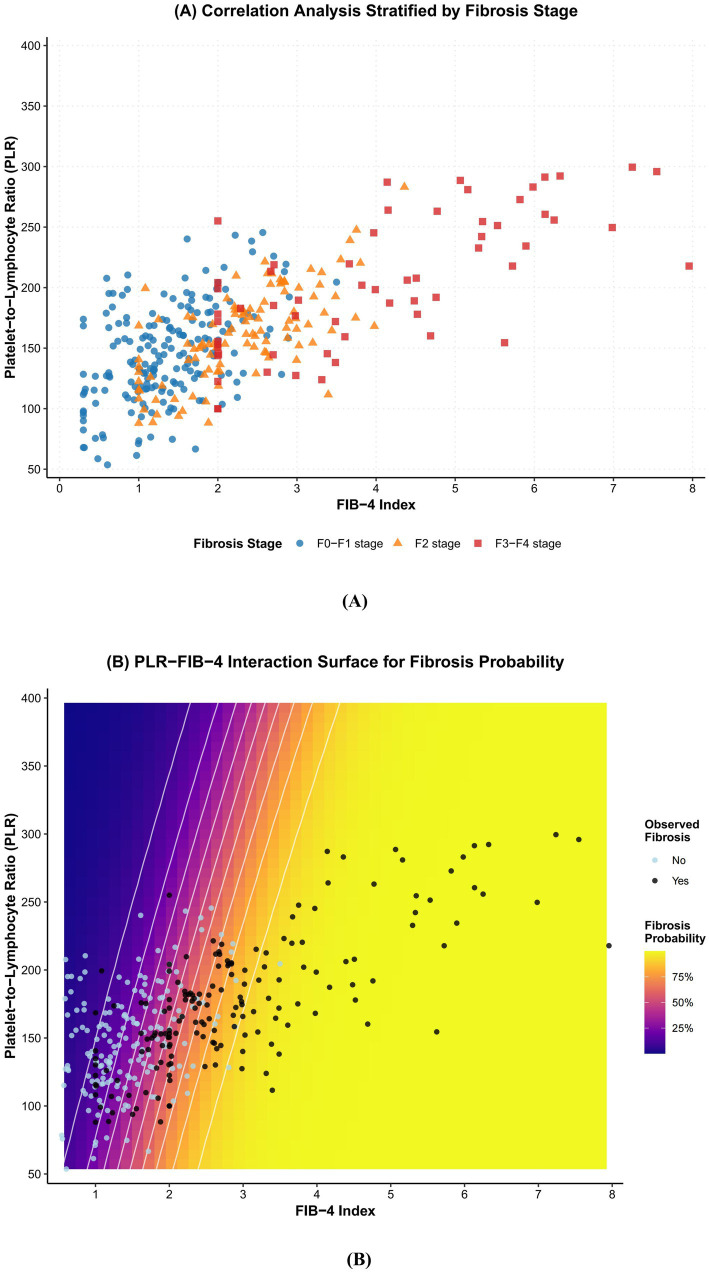
Correlation and interaction analysis. **(A)** Scatterplots showing the association between PLR and FIB-4 index stratified by fibrosis stage. **(B)** Three-dimensional surface plot showing the interaction effect of PLR-FIB-4 on fibrosis probability.

Correlation analysis revealed a significant positive correlation between the PLR and the FIB-4 index across the entire cohort (*r* = 0.542, *p* < 0.001). This positive correlation became progressively stronger with increasing fibrosis severity. In a stratified analysis, the correlation coefficient (*r*) was 0.389 in the F0–F1 group, increased to 0.491 in the F2 group, and strengthened further to 0.623 in the F3–F4 group (all *p* < 0.001; Figure 1).

To systematically evaluate the independent and combined effects of PLR and FIB-4 index in predicting significant fibrosis, four progressively structured logistic regression models were constructed (Table 2). Following the mean-centering of predictors as described in the methods, collinearity diagnostics were performed. All variance inflation factor (VIF) values were well below the threshold of 5, confirming the stability of the models. After adjusting for confounding factors including age, gender, BMI, and diabetes status, the PLR alone model showed that each unit increase in PLR resulted in a 0.9% increase in significant fibrosis risk (OR = 1.009, 95% CI: 1.007–1.012, *p* < 0.001); in the FIB-4 alone model, each unit increase in FIB-4 index resulted in a 178.4% increase in risk (OR = 2.784, 95% CI: 2.208–3.512, *p* < 0.001). In the PLR + FIB-4 combined model, both indicators maintained statistical significance, though with slightly reduced effect sizes, suggesting some degree of information overlap. The key finding was that the regression coefficient for the PLR × FIB-4 product term in the interaction model was 0.0028 (95% CI: 0.0010–0.0046, *p* = 0.003), reaching statistical significance and confirming the presence of positive interaction effects between the two indicators.

**Table 2:** Logistic regression models for predicting significant fibrosis.

Models	Variables	*β*	SE	OR	95%CI	*p*-value
Model 1: PLR	PLR	0.0092	0.0014	1.009	1.007–1.012	<0.001
	Constant term	−2.468	0.241	0.085	0.053–0.136	<0.001
Model 2: FIB-4	FIB-4	1.024	0.118	2.784	2.208–3.512	<0.001
	Constant term	−1.587	0.148	0.205	0.153–0.274	<0.001
Model 3: PLR + FIB-4	PLR	0.0067	0.0015	1.007	1.004–1.010	<0.001
	FIB-4	0.821	0.125	2.273	1.779–2.906	<0.001
	Constant term	−2.891	0.274	0.056	0.033–0.095	<0.001
Model 1: Interaction model	PLR	0.0034	0.0019	1.003	1.000–1.007	0.076
	FIB-4	0.518	0.156	1.679	1.237–2.278	0.001
	PLR × FIB-4	0.0028	0.0009	1.003	1.001–1.005	0.003
	Constant term	−3.524	0.329	0.029	0.015–0.056	<0.001

Models were adjusted for age, sex, BMI, and diabetes status. SE, standard error; OR, odds ratio; CI, confidence interval.

### 3.3 Model diagnostic efficacy evaluation and comparison

To comprehensively evaluate the diagnostic value of different predictive models in identifying significant fibrosis, this study conducted systematic comparative analysis using multiple performance metrics (Table 3). Single-indicator model analysis revealed that the PLR alone model achieved an area under the curve (AUC) of 0.731 (95% CI: 0.679–0.783), with sensitivity and specificity of 69.0 and 68.4%, respectively, and an accuracy of 68.7%. The FIB-4 alone model demonstrated superior performance, achieving an AUC of 0.785 (95% CI: 0.737–0.833), with improved sensitivity of 73.2%, specificity of 74.2%, and accuracy of 73.7%. Combined models further enhanced diagnostic performance. The PLR + FIB-4 additive model achieved an AUC of 0.801 (95% CI: 0.755–0.847), with sensitivity and specificity reaching 75.0 and 76.8%, respectively, and accuracy improving to 75.9%.

**Table 3:** Comparison of diagnostic efficacy for predicting significant fibrosis.

Models	AUC	95%CI	Sensitivity (%)	Specificity (%)	PPV (%)	NPV (%)	Accuracy (%)
PLR	0.731	0.679–0.783	69.0	68.4	64.4	72.6	68.7
FIB-4	0.785	0.737–0.833	73.2	74.2	70.3	77.0	73.7
PLR + FIB-4	0.801	0.755–0.847	75.0	76.8	73.3	78.4	75.9
PLR × FIB-4	0.824	0.778–0.870	78.6	78.9	76.0	81.1	78.8

AUC, Area under the curve; PPV, positive predictive value; NPV, negative predictive value.

**Figure 2:**
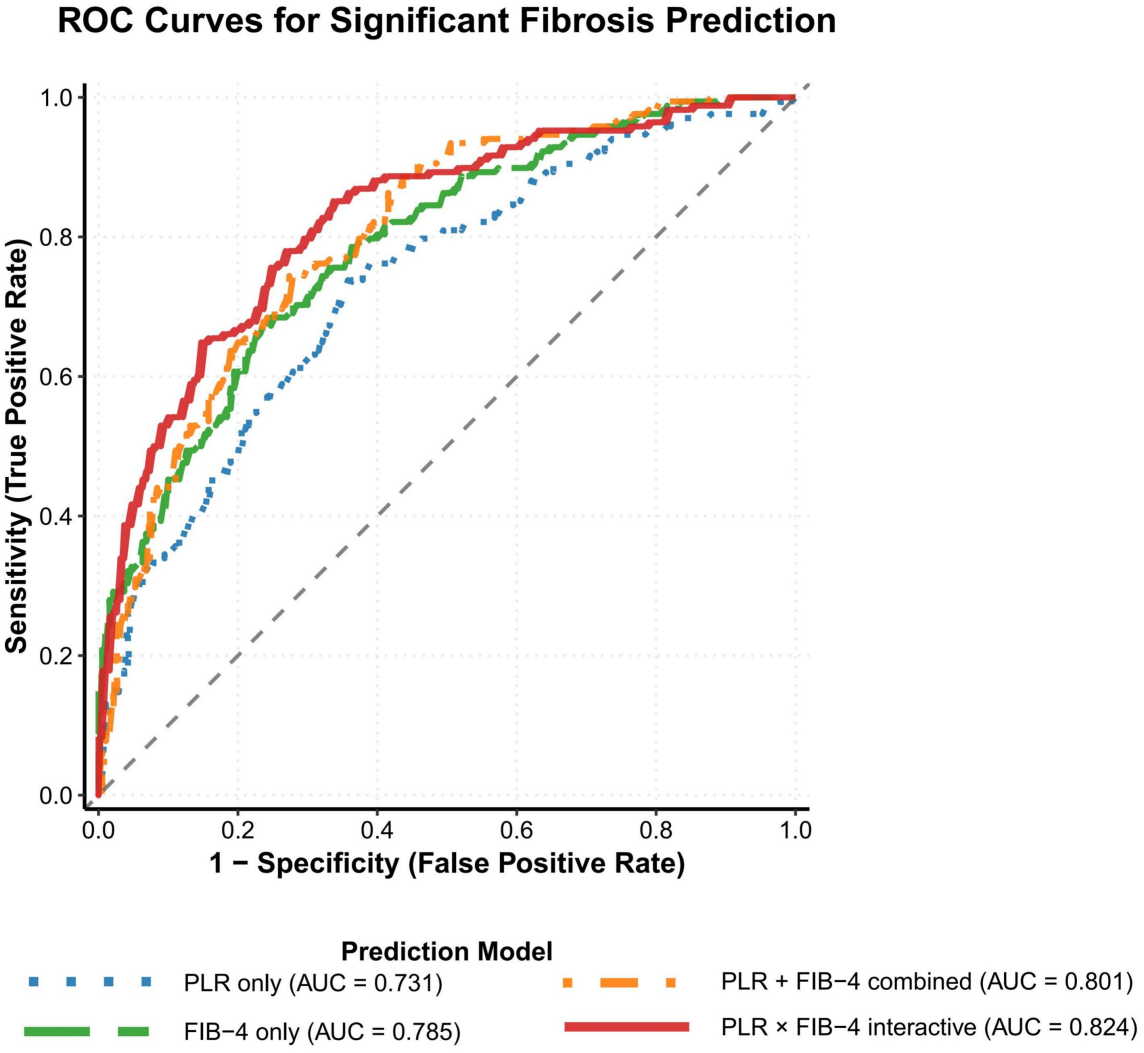
Receiver operating characteristic curves comparing the diagnostic efficacy of different models for predicting significant fibrosis (≥ F2) by ROC curve analysis. The interaction model (red line) showed better AUC than the individual markers and the additive model.

Most importantly, the PLR × FIB-4 interaction model demonstrated optimal diagnostic performance, achieving an AUC of 0.824 (95% CI: 0.778–0.870), with sensitivity and specificity of 78.6 and 78.9%, respectively, positive predictive value of 76.0%, negative predictive value of 81.1%, and overall accuracy of 78.8% (Figure 2). Pairwise comparisons using DeLong test further confirmed the statistical superiority of the interaction model: compared to the PLR alone model, AUC improved by 0.093 (*p* < 0.001); compared to the FIB-4 alone model, AUC improved by 0.039 (*p* = 0.012); and even compared to the PLR + FIB-4 combined model, there was still a significant improvement of 0.023 (*p* = 0.048).

### 3.4 Progressive fibrosis predictive efficacy

In predicting the clinically more critical advanced fibrosis (≥F3 stage), this study further validated the diagnostic value of various predictive models (Table 4). Given that advanced fibrosis patients comprised a relatively small proportion of the study cohort (60 cases, 16.8%), this predictive task placed higher demands on model sensitivity and specificity. Results showed that the PLR alone model achieved an AUC of 0.748 (95% CI: 0.685–0.811) for this predictive task. Although sensitivity was 66.7%, its high negative predictive value (91.5%) indicated good clinical utility for ruling out advanced fibrosis. The FIB-4 alone model demonstrated superior overall performance, with an AUC of 0.808 (95% CI: 0.753–0.863), improved sensitivity of 75.0%, specificity of 79.9%, and negative predictive value further increased to 93.7%.

**Table 4:** Progressive fibrosis (≥ F3) predictive power.

Models	AUC	95%CI	Sensitivity (%)	Specificity (%)	PPV (%)	NPV (%)	Accuracy (%)
PLR	0.748	0.685–0.811	66.7	75.2	35.7	91.5	71.3
FIB-4	0.808	0.753–0.863	75.0	79.9	43.3	93.7	78.7
PLR + FIB-4	0.831	0.780–0.882	78.3	81.5	46.5	94.9	81.2
PLR × FIB-4	0.852	0.803–0.901	80.0	84.6	52.2	95.5	88.1

AUC, Area under the curve; PPV, positive predictive value; NPV, negative predictive value.

**Figure 3:**
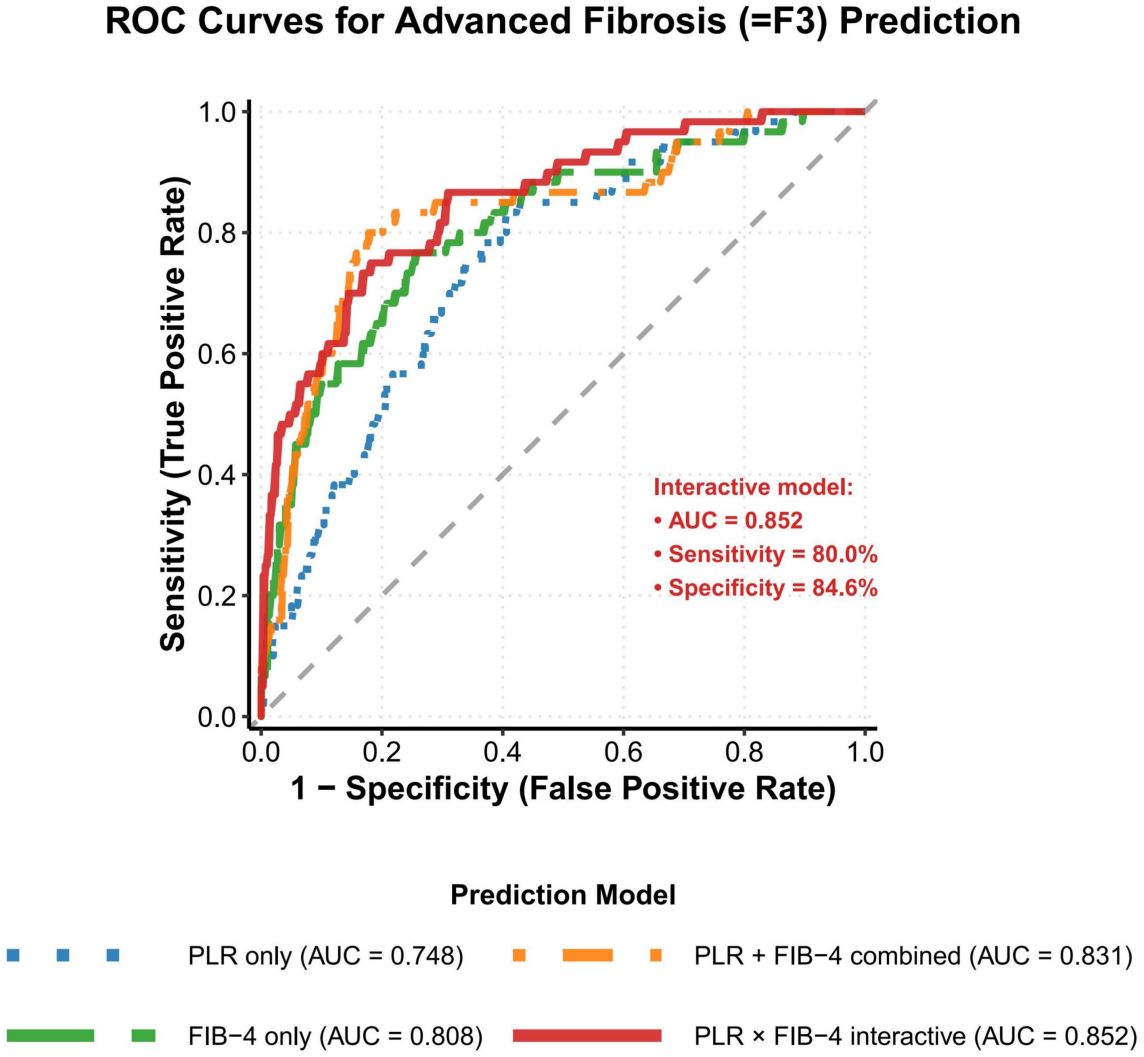
Receiver operating characteristic curves comparing the diagnostic efficacy of different models for predicting progressive fibrosis (≥ F3) by ROC curve analysis. The interaction model (red line) showed better AUC than the individual markers and the additive model.

The PLR + FIB-4 combined model, by integrating information from both indicators, significantly improved predictive performance with an AUC of 0.831 (95% CI: 0.780–0.882), achieving sensitivity and specificity of 78.3 and 81.5%, respectively. More importantly, the PLR × FIB-4 interaction model achieved optimal performance in predicting advanced fibrosis, with an AUC as high as 0.852 (95% CI: 0.803–0.901) (Figure 3). This model demonstrated sensitivity of 80.0% and specificity of 84.6%, achieving an excellent balance between sensitivity and specificity.

Particularly noteworthy is that the interaction model’s positive predictive value reached 52.2%, representing a significant improvement compared to single-indicator models. Meanwhile, the 95.5% negative predictive value ensured high reliability of this model in ruling out advanced fibrosis, providing a solid foundation for clinical decision-making.

### 3.5 Comparison with traditional noninvasive scoring systems

To objectively evaluate the clinical application value of the PLR × FIB-4 interaction model, this study conducted systematic comparisons with three commonly used non-invasive hepatic fibrosis assessment tools in current clinical practice (Table 5). Results demonstrated that the PLR × FIB-4 interaction model comprehensively outperformed existing scoring systems in diagnostic performance. Compared to the APRI-FIB-4 composite score (AUC = 0.772), the interaction model showed an AUC advantage of 0.052, with a net reclassification index (NRI) of 0.142 (*p* = 0.008) and integrated discrimination improvement (IDI) of 0.067 (*p* = 0.003). When compared to the MASLD fibrosis score (AUC = 0.764), the AUC advantage was 0.060, with NRI of 0.156 (*p* = 0.004) and IDI of 0.072 (*p* = 0.003). The most significant difference was observed in comparison with the BARD score, where the PLR × FIB-4 interaction model demonstrated an AUC advantage as high as 0.126, with NRI reaching 0.218 (*p* < 0.001) and IDI of 0.098 (*p* < 0.001).

**Table 5:** Comparison with established noninvasive scores.

Scores	AUC	95%CI	*Vs* interaction model ΔAUC*	NRI	95%CI	*p*-value	IDI	95% CI	*p*-value
APRI-FIB-4 composite^†^	0.772	0.722–0.822	0.052	0.142	0.038–0.246	0.008	0.067	0.023–0.111	0.003
MASLD Fibrosis Score	0.764	0.713–0.815	0.060	0.156	0.049–0.263	0.004	0.072	0.025–0.119	0.003
BARD score	0.698	0.644–0.752	0.126	0.218	0.115–0.321	<0.001	0.098	0.056–0.140	<0.001

*Difference compared to PLR×FIB-4 interaction model; ^†^APRI-FIB-4 composite: (APRI + FIB-4)/2; NRI, net weight classification index; IDI, integrated discrimination improvement index.

### 3.6 Risk stratification analysis based on interaction model

**Figure 4:**
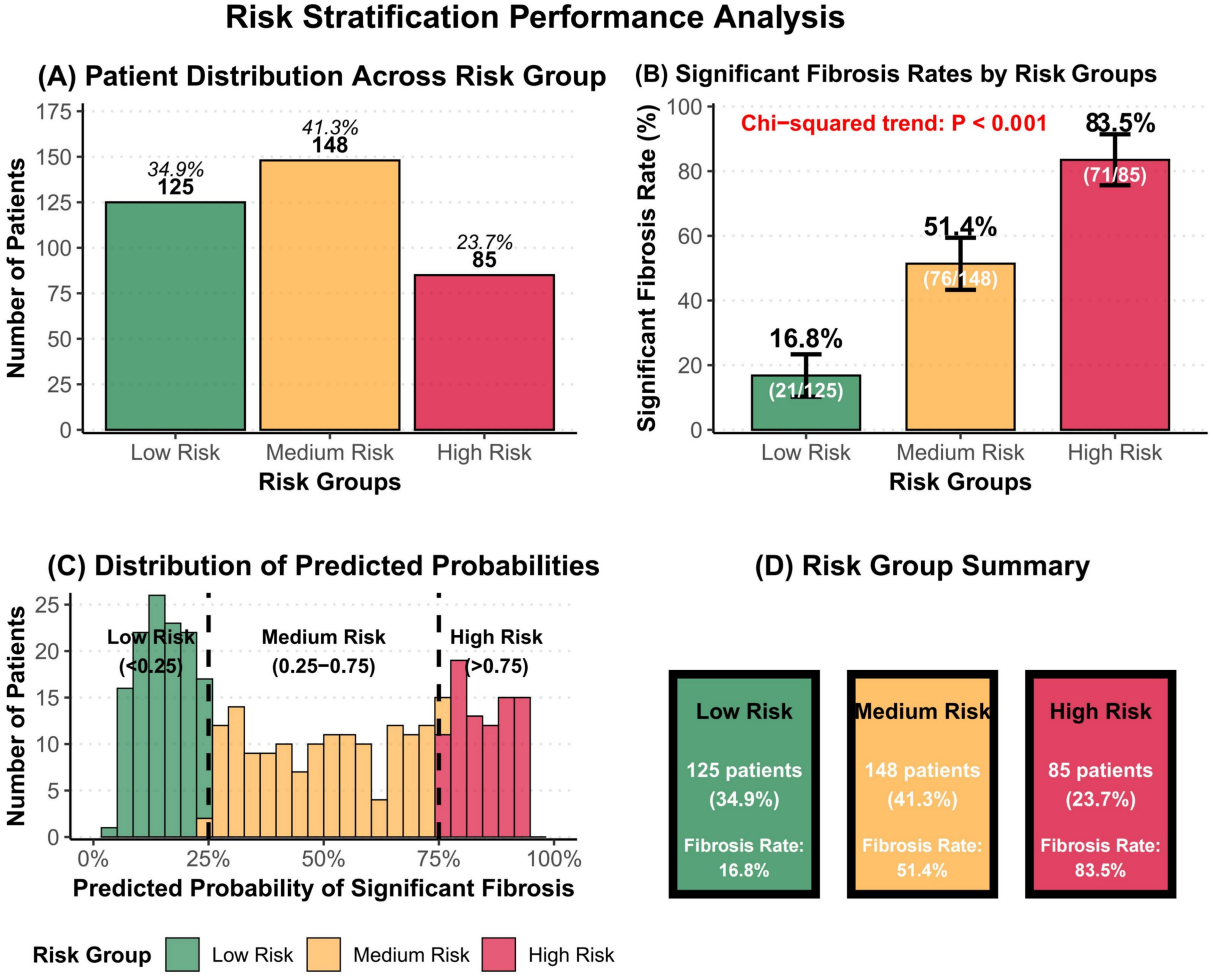
Risk stratification efficacy. **(A)** Distribution of patients in risk groups. **(B)** Incidence of significant fibrosis in different risk groups. **(C)** Predicted probability distribution of significant fibrosis. **(D)** Risk Stratification Summary.

Based on predicted probabilities from the PLR × FIB-4 interaction model, patients were stratified into three distinct risk levels (Table 6). Results demonstrated that this stratification strategy possessed excellent clinical discriminatory value. The low-risk group (predicted probability <0.25) comprised 125 patients (34.9%), with a significant fibrosis occurrence rate of only 16.8% (95% CI: 10.7–24.7%). The intermediate-risk group (predicted probability 0.25–0.75) included 148 patients (41.3%), with a significant fibrosis occurrence rate of 51.4% (95% CI: 42.9–59.8%). The high-risk group (predicted probability >0.75) contained 85 patients (23.7%), with a significant fibrosis occurrence rate as high as 83.5% (95% CI: 74.0–90.6%) (Figure 4). Chi-square test for trend revealed a highly significant gradient difference in significant fibrosis occurrence rates among risk groups (*p* < 0.001).

**Table 6:** Risk stratification efficacy.

Risk groups	Predicted probability	*N* (%)	Marked fibrosis	Occurrence (%)	95%CI
Low risk	<0.25	125 (34.9)	21	16.8	10.7–24.7
Middle risk	0.25–0.75	148 (41.3)	76	51.4	42.9–59.8
High risk	>0.75	85 (23.7)	71	83.5	74.0–90.6

### 3.7 Subgroup analysis validation model stability

To evaluate the applicability of the PLR × FIB-4 interaction model across different patient populations, this study conducted systematic subgroup analyses according to key clinical characteristics (Table 7). Age-stratified analysis revealed AUCs of 0.819 and 0.828 for younger patients (<50 years) and middle-aged to elderly patients (≥50 years), respectively, with no statistically significant difference (*p* = 0.782). In gender subgroups, male and female patients demonstrated AUCs of 0.831 and 0.814, respectively (*p* = 0.531). BMI stratification showed AUCs of 0.816 for the non-obese group (<30 kg/m^2^) and 0.841 for the obese group (≥30 kg/m^2^) (*p* = 0.463). Diabetes status stratification revealed AUCs of 0.809 for the non-diabetic group and 0.845 for the diabetic group (*p* = 0.286). All *p*-values for AUC comparisons between subgroups exceeded 0.05, indicating that the predictive performance of the PLR × FIB-4 interaction model was not significantly influenced by age, gender, BMI, or diabetes status, demonstrating excellent stability and generalizability.

**Table 7:** Subgroup analysis of interaction model power.

Subgroup	*n*	AUC	95%CI	*p* value*
Age				
<50	158	0.819	0.750–0.888	0.782
≥50	200	0.828	0.769–0.887	
Sex				
Male	208	0.831	0.774–0.888	0.531
Female	150	0.814	0.745–0.883	
BMI				
<30	242	0.816	0.760–0.872	0.463
≥30	116	0.841	0.766–0.916	
Diabetes				
No	216	0.809	0.747–0.871	0.286
Yes	142	0.845	0.779–0.911	

*p-values for AUC comparisons between subgroups.

### 3.8 Assessment of clinical decision-making effectiveness

**Figure 5:**
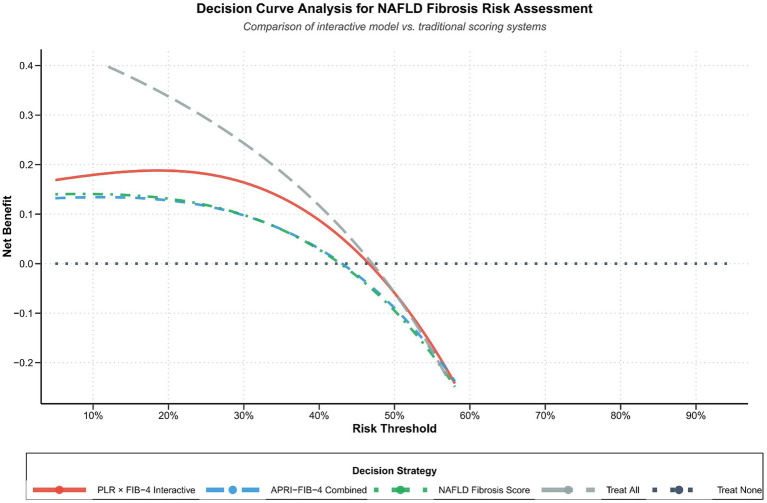
Decision curve analysis compares the net benefit analysis of the interaction model with the traditional score at different probability thresholds and shows the superior clinical utility of the interaction method.

Decision curve analysis revealed that the PLR × FIB-4 interaction model provided significantly higher clinical net benefit compared to existing scoring systems within the key probability threshold range (0.01–0.58) relevant to clinical practice. At the most commonly used probability threshold of 0.30, the interaction model demonstrated clear advantages: net benefit was 0.089 higher than the APRI-FIB-4 composite score and 0.086 higher than the MASLD fibrosis score. These results indicate that adopting the interaction model for clinical decision-making can provide correct diagnostic and therapeutic benefits to more patients at the same diagnostic threshold, while reducing unnecessary overdiagnosis and overtreatment (Figure 5).

### 3.9 Model stability internal validation

**Figure 6:**
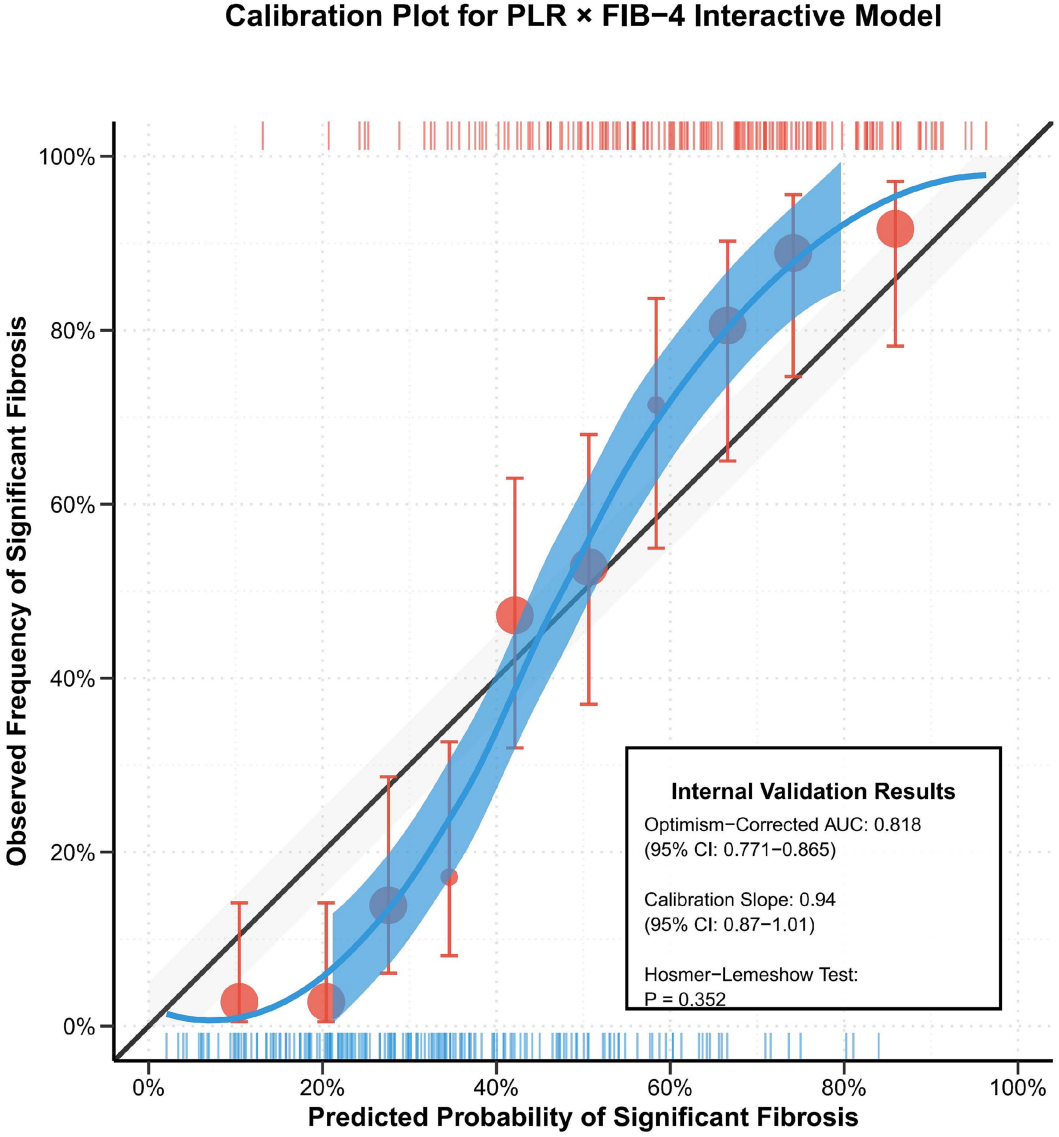
Calibration plot showing the consistency of the predicted probability of the interaction model with the observed fibrosis incidence, with 95% confidence intervals and perfect calibration lines.

To ensure the reliability of the study results, rigorous internal validation of the interaction model was performed using Bootstrap resampling methodology (*n* = 1,000 iterations). Validation results demonstrated excellent model stability: the optimism-corrected AUC was 0.818 (95% CI: 0.771–0.865), showing high consistency with the original model performance; the calibration slope was 0.94 (95% CI: 0.87–1.01), approaching the ideal value of 1.0, indicating excellent agreement between predicted probabilities and actual occurrence rates; the Hosmer-Lemeshow goodness-of-fit test yielded *p* = 0.352, demonstrating good model calibration and reliable predictive accuracy. These validation results fully confirm the statistical robustness and clinical application value of the PLR × FIB-4 interaction model (Figure 6).

### 3.10 Sensitivity analyses

To test the robustness of the PLR × FIB-4 interaction model, several sensitivity analyses were performed, with results summarized in Table 8. First, in the subgroup of 115 patients with normal ALT levels (≤40 U/L), the model maintained excellent diagnostic efficacy for significant fibrosis, achieving an AUC of 0.815 (95% CI: 0.738–0.892). This performance was not statistically different from its efficacy in patients with elevated ALT (AUC = 0.830, *p* = 0.681). Second, the model’s diagnostic accuracy was shown to increase with fibrosis severity. The AUC for predicting significant fibrosis (≥F2) was 0.824, which increased to 0.852 for advanced fibrosis (≥F3), and further to 0.903 for cirrhosis (F4), demonstrating its strong discriminative power across the disease spectrum. Third, we assessed the influence of common platelet-affecting comorbidities by using diabetes as a proxy. As previously detailed in the subgroup analysis (Section 3.7), the model’s performance was robust and did not differ significantly between diabetic and non-diabetic patients. Finally, the interval between blood draw and liver biopsy did not impact the model’s performance. The AUC for patients with a short interval (≤30 days, *n* = 212) was 0.829, which was nearly identical to the AUC for those with a longer interval (31–180 days, *n* = 146) at 0.818 (*p* = 0.745). These analyses confirm the stability and reliability of the interaction model across various clinically relevant scenarios.

**Table 8:** Results of sensitivity analyses for the PLR × FIB-4 interaction model.

Sensitivity analysis	Subgroup	*N*	AUC (95% CI)	*p*-value for AUC comparison
Normal ALT status (For predicting ≥F2 Fibrosis)	ALT ≤ 40 U/L	115	0.815 (0.738–0.892)	0.681
	ALT > 40 U/L	243	0.830 (0.779–0.881)	
Performance across stages (Full Cohort)	Predicts Significant Fibrosis (≥F2)	358	0.824 (0.778–0.870)	N/A
	Predicts Advanced Fibrosis (≥F3)	358	0.852 (0.803–0.901)	N/A
	Predicts Cirrhosis (F4)	358	0.903 (0.844–0.962)	N/A
Biopsy-to-Lab Interval (For predicting ≥F2 Fibrosis)	≤ 30 days	212	0.829 (0.773–0.885)	0.745
	31–180 days	146	0.818 (0.750–0.886)	

ALT, Alanine Aminotransferase; AUC, Area Under the Receiver Operating Characteristic Curve; CI, Confidence Interval; N, number of patients. *p*-values are for the comparison of AUCs between the respective subgroups.

## 4 Discussion

This study represents the first systematic evaluation of the diagnostic value of the interaction between PLR and FIB-4 index in hepatic fibrosis risk stratification among MASLD patients. Results demonstrate that the PLR × FIB-4 interaction model significantly outperforms traditional single indicators and composite scoring systems. The interaction model exhibited excellent diagnostic efficacy in predicting both significant fibrosis and advanced fibrosis, providing a novel and effective tool for clinical non-invasive hepatic fibrosis assessment.

Our study revealed a significant positive interaction between the PLR and FIB-4 index, a finding with crucial pathophysiological implications that suggest a synergistic effect rather than a simple additive one. The development and progression of hepatic fibrosis is not a linear process but a vicious cycle involving inflammatory responses, hepatocyte injury, platelet dynamics, and immune dysregulation (23). The synergy between PLR and FIB-4 can be understood through a multi-layered mechanistic feedback loop (24, 25): First, chronic inflammation acts as a common upstream driver. In MASLD, metabolic stress triggers the release of pro-inflammatory cytokines such as Interleukin-6 (IL-6) and Tumor Necrosis Factor-alpha (TNF-*α*) from both inflamed adipose tissue and hepatic immune cells (26). These cytokines exert dual effects: they directly promote the activation of hepatic stellate cells and collagen synthesis, driving the progression of fibrosis (a key component reflected in the FIB-4 score), and they also modulate hematopoiesis in the bone marrow, affecting platelet production and lymphocyte function and survival, which are the core components of the PLR (27). Thus, the initial inflammatory state simultaneously lays the groundwork for changes in both indicators. Second, the progression of hepatic fibrosis acts as an amplifier of systemic hematological disturbances. As liver scarring advances, leading to increased intrahepatic resistance, portal hypertension develops. A major consequence of portal hypertension is congestive splenomegaly and subsequent hypersplenism. The enlarged spleen actively sequesters and destroys platelets and lymphocytes, leading to thrombocytopenia and lymphopenia (28). These changes are central to the calculation of both the FIB-4 index (where platelet count is a key denominator) and the PLR. The synergy of the interaction term PLR × FIB-4 arises from the convergence of these two processes into a positive feedback loop. In a patient with a high underlying inflammatory state (reflected by an elevated PLR), the advancement of fibrosis (and a rising FIB-4 score) triggers hypersplenism. The resulting sequestration of platelets dramatically reduces their count, which non-linearly amplifies the FIB-4 value. Concurrently, the reduction in both platelets and lymphocytes can further elevate the PLR. This means the structural progression of the disease (fibrosis) actively worsens the hematological and inflammatory indicators, creating a multiplicative, rather than additive, effect on the risk score. The interaction term, therefore, does not merely combine two risk factors; it mathematically captures the potent feedback loop where inflammation initiates fibrosis, and established fibrosis, in turn, amplifies the very systemic signals of inflammation and platelet dysregulation (28, 29). Furthermore, age-related immunosenescence may modulate this entire feedback loop, explaining why the interaction model maintains robust performance across different age strata, effectively correcting for biases where single indices like FIB-4 may fail (30).

The significant advantages of the interaction model in diagnostic efficacy are manifested at multiple levels. First, the AUC of 0.824 significantly exceeds that of currently widely applied clinical tools such as APRI (AUC approximately 0.65–0.75) and NFS (AUC approximately 0.70–0.80) (31). More importantly, the interaction model achieves excellent balance between sensitivity and specificity, which holds crucial clinical decision-making significance. High sensitivity (78.6%) ensures effective identification of patients with significant fibrosis, preventing disease progression due to missed diagnoses, while high specificity (78.9%) reduces false-positive rates, avoiding unnecessary further examinations and patient anxiety (32). Particularly in predicting advanced fibrosis, the interaction model achieves an AUC as high as 0.852 with a negative predictive value of 95.5%, indicating extremely high reliability in excluding advanced fibrosis and effectively reducing the need for liver biopsy (33).

A key strength of our study is the development of a clear, three-tier risk stratification strategy. It is crucial to position this PLR × FIB-4 interaction model not as a replacement for specialized tests like transient elastography (TE), but as a highly effective, cost-free, first-line triage tool to complement them. Based on our findings, we propose the following clinical decision-making pathway, which could significantly enhance diagnostic efficiency and resource allocation (34): Low-Risk Group (Predicted Probability <0.25): This group constituted 34.9% of our cohort and had a low actual rate of significant fibrosis (16.8%), corresponding to a high Negative Predictive Value (NPV) of 83.2%. For these patients, the model provides a high degree of confidence to rule out significant fibrosis. The clinical action would be reassurance, continued lifestyle management, and routine monitoring in a primary care setting, thereby safely avoiding unnecessary referrals and costly second-line examinations. High-Risk Group (Predicted Probability >0.75): This group comprised 23.7% of patients and demonstrated a very high rate of significant fibrosis (83.5%), conferring a strong Positive Predictive Value (PPV). These individuals should be considered high-priority for an urgent referral to a hepatologist or gastroenterologist. They should undergo confirmatory second-line testing (e.g., TE, MRE) and be considered for liver biopsy and aggressive management strategies. Intermediate-Risk Group (Predicted Probability 0.25–0.75): This “gray zone” represented the largest segment of our cohort (41.3%), with a significant fibrosis rate of 51.4%. This is precisely the population where diagnostic uncertainty is highest. The clinical action for this group is to proceed with second-line non-invasive testing (e.g., TE) to further clarify their fibrosis stage. Our model’s main value lies in efficiently identifying this group, ensuring that TE and specialist resources are directed toward patients who need it most, rather than being used unscreened on the entire at-risk population. From a health economics perspective, implementing this pathway allows for the strategic use of routine blood parameters to manage the vast majority of MASLD patients, reserving more expensive technologies for the subset with the highest pre-test probability or diagnostic uncertainty. This targeted approach optimizes clinical workflow and holds the potential for substantial cost savings (35).

Subgroup analysis results demonstrated that the interaction model maintained stable predictive performance across patient populations of different ages, genders, BMI categories, and diabetes status, a finding of significant clinical utility (36). Previous studies have shown that traditional non-invasive scoring systems are often influenced by demographic characteristics and comorbidities, performing poorly in specific subgroups (37). For example, the FIB-4 index may underestimate fibrosis severity in younger patients while potentially overestimating it in elderly patients (38). In our study, AUC differences among subgroups were not statistically significant, indicating that the interaction between PLR and FIB-4 effectively corrects these potential biases and improves model generalizability.

Bootstrap internal validation further confirmed model robustness. The optimism-corrected AUC (0.818) showed high consistency with the original model, and the calibration slope approached the ideal value of 1.0, indicating excellent agreement between predicted probabilities and actual occurrence rates (39). Such good calibration is crucial for clinical application, as it ensures the accuracy of predicted probabilities and provides reliable risk assessment information for physicians and patients (40).

This study conducted comprehensive comparisons between the interaction model and three commonly used clinical non-invasive scores, with results showing significant advantages for the interaction model in all comparisons. Compared to the APRI-FIB-4 composite score, the interaction model achieved an NRI of 0.142 and IDI of 0.067, meaning that among 100 patients, the interaction model could correctly reclassify approximately 14 patients and significantly improve discriminative ability for risk prediction (41). More remarkably, compared to the BARD score, the interaction model demonstrated an AUC advantage as high as 0.126 with an NRI of 0.218—such substantial improvement is extremely rare in biomarker research (42). This result may be related to the fact that the BARD score is primarily based on simple clinical parameters (BMI, AST/ALT ratio, diabetes), while the interaction model, by integrating multidimensional information on inflammation and fibrosis, can more accurately reflect the pathophysiological processes of the disease (43).

Decision curve analysis provided intuitive assessment of the model’s clinical application value. At the 0.30 probability threshold, the interaction model provided significantly higher net benefit compared to traditional scores, indicating that adopting the interaction model for decision-making can provide correct diagnostic and therapeutic benefits to more patients (44). From a clinical practice perspective, this improvement in net benefit can translate into reduced unnecessary liver biopsies, optimized patient triage, and improved resource allocation (45). Particularly in the context of continuously rising MASLD prevalence, non-invasive tools capable of accurately identifying high-risk patients hold important public health significance (46).

This study has several limitations that must be carefully considered. The primary and most significant limitation is its design as a retrospective study conducted within a specific regional cohort, coupled with the lack of external validation. This issue is further compounded by the nature of a biopsy-confirmed cohort, which inherently selects for patients from tertiary referral centers. The high prevalence of significant fibrosis (46.9%) and advanced fibrosis (16.8%) in our study is indicative of this selection bias. This may lead to an overestimation of the model’s diagnostic accuracy compared to its potential performance in a primary care or general population setting with a lower disease prevalence. Therefore, while the model was rigorously internally validated, its generalizability remains unconfirmed. We must strongly emphasize that before this model can be recommended for clinical implementation, its performance must be prospectively validated in large, independent, and geographically diverse multi-center external cohorts, including those from primary care settings. Secondly, our study focused on a static prediction based on a single time-point assessment. The cross-sectional nature of the data prevents us from drawing conclusions about the model’s utility in monitoring disease progression or predicting dynamic changes in fibrosis over time. Future longitudinal studies are needed to explore the impact of changes in PLR and FIB-4 on predictive performance and to assess the model’s value in tracking treatment response. Finally, although our exclusion criteria were strict, the retrospective collection of data may still be subject to unmeasured confounding variables. Prospective studies would provide a higher level of evidence by allowing for more controlled data collection. In conclusion, while our study introduces a novel and promising interaction model, its current utility is as a proof-of-concept for high-risk populations. Its translation into a reliable tool for widespread clinical practice is entirely contingent upon addressing these limitations, most critically through rigorous external validation across a spectrum of clinical settings.

This study demonstrates that PLR and FIB-4 exhibit significant interactive effects in MASLD fibrosis prediction. The interactive model provides superior diagnostic performance compared to traditional composite scores, with clinically meaningful improvements in risk stratification. These findings support the clinical utility of considering biomarker interactions for enhanced non-invasive fibrosis assessment in MASLD patients. However, external validation in larger, multi-center cohorts is needed before widespread clinical implementation.

## Data Availability

The original contributions presented in the study are included in the article/supplementary material, further inquiries can be directed to the corresponding author.
